# Antiviral Activity of Tannic Acid Modified Silver Nanoparticles: Potential to Activate Immune Response in Herpes Genitalis

**DOI:** 10.3390/v10100524

**Published:** 2018-09-26

**Authors:** Piotr Orłowski, Andrzej Kowalczyk, Emilia Tomaszewska, Katarzyna Ranoszek-Soliwoda, Agnieszka Węgrzyn, Jakub Grzesiak, Grzegorz Celichowski, Jarosław Grobelny, Kristina Eriksson, Malgorzata Krzyzowska

**Affiliations:** 1 Military Institute of Hygiene and Epidemiology, 01-163 Warsaw, Poland; piotr.r.orlowski@gmail.com; 2 PORT Polish Center for Technology Development, 54-066 Wroclaw, Poland; andrzej.kowalczyk@port.org.pl (A.K.); agnieszka.wegrzyn@port.org.pl (A.W.); grzesiak.kuba@gmail.com (J.G.); 3 Department of Materials Technology and Chemistry, Faculty of Chemistry, University of Lodz, 90-236 Lodz, Poland; emilia.tomaszewska@chemia.uni.lodz.pl (E.T.); katarzyna.soliwoda@chemia.uni.lodz.pl (K.R.-S.); gcelichowski@uni.lodz.pl (G.C.); jgrobel@uni.lodz.pl (J.G.); 4 Department of Rheumatology & Inflammation research, Gothenburg University, 405 30 Gothenburg, Sweden; kristina.eriksson@microbio.gu.se

**Keywords:** tannic acid, silver nanoparticle, HSV-2

## Abstract

(1) Background: Tannic acid is a plant-derived polyphenol showing antiviral activity mainly because of an interference with the viral adsorption. In this work, we tested whether the modification of silver nanoparticles with tannic acid (TA-AgNPs) can provide a microbicide with additional adjuvant properties to treat genital herpes infection. (2) Methods: The mouse model of the vaginal herpes simplex virus 2 (HSV-2) infection was used to test immune responses after treatment of the primary infection with TA-AgNPs, and later, after a re-challenge with the virus. (3) Results: The mice treated intravaginally with TA-AgNPs showed better clinical scores and lower virus titers in the vaginal tissues soon after treatment. Following a re-challenge, the vaginal tissues treated with TA-AgNPs showed a significant increase in the percentages of IFN-gamma+ CD8+ T-cells, activated B cells, and plasma cells, while the spleens contained significantly higher percentages of IFN-gamma+ NK cells and effector-memory CD8+ T cells in comparison to NaCl-treated group. TA-AgNPs-treated animals also showed significantly better titers of anti-HSV-2 neutralization antibodies in sera; and (4) Conclusions: Our findings suggest that TA-AgNPs sized 33 nm can be an effective anti-viral microbicide to be applied upon the mucosal tissues with additional adjuvant properties enhancing an anti-HSV-2 immune response following secondary challenge.

## 1. Introduction

Herpes simplex virus 2 (HSV-2) is one of the most frequent genital ulcerative diseases worldwide. In 2012, an estimated 417 million people aged 15–49 (11%) were living with HSV-2 infection, of whom, approximately 50% were women [1]. After establishing latency in the sacral ganglia, HSV-2 reactivates, leading to intermittent shedding of the virus in the genital tract [2]. The standard clinical treatment of symptomatic HSV-2 infections requires the use of nucleoside analogues, such as acyclovir (ACV), famciclovir (FAM), and valacyclovir (VCV), which target viral DNA polymerase [3,4]. Nucleoside analogues are used to treat both primary and recurrent infections. However, to date, none of them are effective in eliminating an established latent infection, and their prolonged clinical use in immunocompromised patients may lead to the development of antiviral-resistant virus strains [3]. There is no vaccine protecting from HSV-2 infection to date, although several experimental models have been proposed [5].

HSV-2 causes a disruption to the integrity of the vaginal mucosa, which heightens the risk of acquiring other sexually transmitted diseases (STDs), such as human immunodeficiency virus type 1 (HIV-1) [6,7]. Recurrent herpes infections are also a problem in malignancies—the recurrent infections influence the arrangement of medical procedures [8]. Therefore, there is a need to develop an effective anti-herpes microbicide that can to the bind cell-free virus, blocking its further spread and inactivating free virions. At the same time, it should be safe and non-toxic when applied topically on the skin and epithelial mucosal tissue.

Tannins are water-soluble phenolic compounds, synthesized and accumulated by higher plants as secondary metabolic products. Tannic acid is a plant-derived hydrolysable tannin with five digalloyl units connected with one glucose molecule (C_76_H_52_O_46_, 1701 Da). The anti-oxidative, anti-inflammatory, and antiviral properties of tannic acid have been described [9]. Lin et al. (2011) demonstrated that by interacting with HSV-1 glycoproteins, the hydrolysable tannins isolated from fruits of the *Terminalia chebula* block the attachment, entry, and cell-to-cell spread of the virus [10].

There are also reports showing the antiviral activities of tannic acid against other viruses, and its action is generally thought to rely upon interference, with viral adsorption to the host cell membrane (e.g., noroviruses, the influenza A virus, and papilloma viruses) [11,12].

Recently, biologically active phytomolecules that are present in plant extracts have been successfully applied in the green synthesis of silver nanoparticles (AgNPs), acting as functionalizing ligands [13]. Green chemistry is the utilization of principles that will help to reduce or eliminate the use or generation of hazardous substances [14]. Silver nanoparticles (AgNPs) synthesized with plant extracts are a promising source of new antiviral agents, because of their multi-targeting mechanism of action. Silver nanoparticles have been demonstrated to exert an antiviral activity against viruses such as hepatitis B virus [15], HIV-1 [16], herpes simplex virus type 1 [17], and influenza virus [18].

We have previously described the anti-HSV-2 activity of tannic acid modified AgNPs (TA-AgNPs) by using both in vitro and in vivo models [19,20]. The mechanisms of the antiviral action of TA-AgNPs includ blocking the virus attachment and entry, as well as the induction of anti-viral cytokine and chemokine production. The profile of cytokines and chemokines produced during HSV-2 infection demonstrated time- and size-related differences, when treatment with each type of TA-AgNPs was used [19]. We also found that TA-AgNPs were good stimulators of dendritic cell (DC) maturation and TLR9 expression in vitro. HSV-2 pre-incubated with TA-AgNPs caused the up-regulation of MHC II and CD86, and the down-regulation of the CD80 expression on DCs. These effects strongly supported the notion that TA-AgNPs can overcome the inhibition of DC maturation by a live or inactivated virus. Down-regulation of the CD40 expression in HSV-2 infected DCs was not observed when HSV-2 was treated with TA-NPs sized >30 nm [21]. This indicates that tannic acid-modified AgNPs may act, not only as an effective microbicide, but also as a novel class of nano-adjuvants, and can help to overcome the HSV-2-induced suppression of immune activation. However, little is known about the role of tannic acid modified AgNPs as nano-adjuvants in vivo, when applied to treat a primary infection or re-current infection. This is of great importance, as latently infected patients typically experience two to three recurrent infections per year.

We hypothesize that the combination of tannic acid and silver nanoparticles consists an effective anti-viral microbicide to be applied on the mucosal tissues, with additional adjuvant properties boosting the anti-viral response, not only during the primary infection, but also later upon recurrent infection. In this study, we used TA-AgNPs of 33 nm in size, which show moderate toxicity in vitro and do not induce inflammatory responses, to treat the primary HSV-2 intravaginal infection in a mouse model. We tested for the mucosal immunity early after the primary infection, and later upon re-challenge with HSV-2. We showed that TA-AgNPs enhance an anti-HSV-2 immune response following a secondary challenge, which is important in the context of the latent characteristic of herpes virus infections. Our study discusses both the cellular and humoral aspects of the early and late anti-HSV-2 response upon treatment with TA-AgNPs.

## 2. Materials and Methods

### 2.1. Synthesis of TA-AgNPs

Silver nitrate AgNO_3_, (Sigma-Aldrich, St. Louis, MO, USA, 99.999% metal basis), sodium citrate C_6_H_5_Na_3_O_7_·2H_2_O (Sigma-Aldrich, ≥99%), and tannic acid C_76_H_52_O_46_ (Fluka, Seelze, Germany) were used for all of the syntheses, without additional purification. A Deionizer Millipore Simplicity UV system (Millipore, Merck, Warsaw, Poland) was the source of water for all of the syntheses. AgNPs (100 ppm) sized 33 nm were synthesized by the chemical reduction method in water. This synthesis procedure was described previously [19,20], and it was as follows: a reducing mixture was added to the aqueous solution of silver nitrate, which was heated to boiling point under a reflux. After a few minutes, the solution changed to a brownish color, indicating the formation of AgNPs. The mixture was stirred for an additional 15 min. The TA-AgNPs colloid was protected from light and filtered through a 0.1 μm polyvinylidene fluoride (PVDF) membrane, before its further use in biological tests. The synthesized nanoparticles underwent characterization using scanning transmission electron microscopy (STEM), dynamic light scattering (DLS), and UV-VIS spectroscopy, as described previously [22].

### 2.2. Virus Preparation

HSV-2 strain 333 [23] was propagated in African green monkey kidney cells (Vero). The virus titers (PFU/mL) were determined using Vero cells for the plaque assay.

### 2.3. Virus-AgNPs’ Interaction Analysis

To perform an electron microscopic analysis of the HSV-2–AgNP interaction, a virus-nanoparticle suspension, incubated together for 30 min, as described previously [19], was fixed overnight in 2.5% glutaraldehyde with the quick addition of 25% glutaraldehyde to the solution, at a proportion of 1:9.

Next, a 50 µL droplet of the so-prepared suspension was put onto the Formvar-coated copper TEM grid for 15 min, in order to allow sedimentation. Subsequently, the liquid was blotted with a paper filter, and three droplets of 100 nm-filtered distilled water were placed onto the specimen. Next, a 1% uranyl acetate aqueous solution droplet was placed on the specimen for 30 s, followed by another triple washing in distilled water. The prepared specimen was undertaken for STEM and TEM analyses. For STEM, a blotted and dried specimen was loaded onto a STEM carrier and analyzed by means of an Auriga 60 (Zeiss, Oberkochen, Germany) scanning electron microscope with a STEM detector, at 30 kV of acceleration voltage. An oriented dark field (ODF) mode with inverted LUT was used for the observations. In order to confirm the identity of the observed objects, a line-scan energy-dispersive X-ray analysis was performed using the Oxford EDX detector. For TEM, a specimen was loaded onto a TEM carrier, and was analyzed by means of a Tecnai X2 (Fei) transmission electron microscope, at 200 kV of acceleration voltage using bright field mode.

### 2.4. Genital HSV-2 Infection and Virus Challenge

Female C57BL/6 mice, between six to eight weeks old, were injected subcutaneously with 2.0 mg/mouse of medroxyprogesterone (Depo-Provera; Upjohn Puurs, Puurs, Belgium) in 200 µL of PBS. Five days later, the mice were anesthetized and inoculated intravaginally with 20 μL of a virus solution, containing 10^3^ PFU of HSV-2 strain 333 in 0.9% NaCl [18]. The protocol was approved by the local committees on the ethics of animal experiments in Warsaw, Poland (35/2011 and 58/2013) and in Gothenburg, Sweden (12/2014). Vaginal washings were performed at 6, 24, and 48 h after infection by repeated (10 times) pipetting with 100 μL of TA-AgNPs diluted in 0.9% NaCl at 5 µg/mL, or 0.9% NaCl. The illness was scored according to the following scale: no signs = 0; slight inflammation of anogenital area = 1; gross inflammation = 2; gross inflammation and hair loss = 3; and gross inflammation, ulceration, and neurological signs = 4. The animal scores were averaged in each group in order to obtain a single representative value. Thirty days later, the mice from both of the treated groups were infected again with the same virus dose. The mice were sacrificed at day 8 post infection, or at day 10 after virus re-challenge. The spinal cords, vaginas, spleens, and blood were collected for further tests.

### 2.5. Virus Titration

The total DNA was isolated from the spinal cords and vaginal tissues preserved in RNAlater (Thermo Fisher Scientific, Waltham, MA, USA) using an RNA/DNA Extracol kit (Eurx, Gdansk, Poland). HSV-2 was detected using a HSV-2 probe labeled with FAM in a real-time PCR instrument, the Stratagene MX4000 Real-Time qPCR System (Agilent Technologies, Santa Clara, CA, USA), as described by Namvar et al. in 2005 [24]. A plasmid vector, pCR 2.1, containing envelope glycoprotein (gB) gene fragment was constructed and purified by the Institute of Biochemistry and Biophysics, Polish Academy of Sciences (Warsaw, Poland). The standard curve analysis was based on Ct values and the serial of 10-fold dilutions of the plasmid standard with an initial concentration of 2.62 × 10^6^ HSV-2 genome copy numbers per reaction. A standard curve was included in each PCR run. The amplification efficiency (E) was calculated from the standard curves, using the formula E = 10(−1/a) − 1, where a is the slope. Data are expressed as the HSV-2 copy number per ng of the total DNA in the tissue.

### 2.6. Flow Cytometry Phenotypic Analysis

Cell suspensions from the vaginal tissues were prepared as follows: The tissues were cut into small pieces and treated with Liberase TL (Roche, Indianapolis, IN, USA) in a MEM medium at 37 °C for 40 min. The treated tissues were pressed through a 70 µm cell strainer, and washed in PBS/1% FBS. The cell suspensions were pretreated with the Fc receptors block, rat anti-CD16/32 antibody (2.4G2) (BD Biosciences, Franklin Lakes, NJ, USA), according to the manufacturer’s protocol. The T cells were detected using rat anti-CD3e-FITC (145-2C11, eBioscience, Santa Clara, CA, USA), rat anti-CD4-PE or BV421 (RM4-5, BD Biosciences), and rat anti-CD8-PE or BV421 (53-6.7., BD Biosciences) antibodies, and the NK cells were detected using the rat anti-NK1.1-APC (PK136, BD Biosciences) antibody. The expression of CD69 was detected using the hamster anti-CD69-APC antibody (H1.2F3, eBioscience). The dendritic cells were detected using the hamster anti-CD11c-APC (HL3, eBioscience), rat anti-F4/80-FITC (BM8, eBioscience), and rat anti-I-A-PE (M5/114.15.2, eBioscience) antibodies, and the monocytes were stained with rat anti-Gr-1-PE (RB6-8C5, BD Biosciences) and rat anti-CD11b-APC (M1/70, BD Biosciences). The B cells were detected using ant-CD45R-APC (B220) (RA3-6B2, eBioscience), anti-CD27-PE (LG.7F9, eBioscience), anti-CD80-PE-Cyanine7 (16-10A1, eBioscience), anti-IgD-FITC (11-26c (11-26), eBioscience), and anti-CD138-PE (300506, eBioscience). Following the immunolabelling for the extracellular markers, the cells were fixed with a Perm/Wash buffer (BD Bioscience) and were incubated with anti-IFN-γ APC-Cy7 (eBioscience, clone-XMG1.2). For all of the phenotyping, the rat immunoglobulin G (IgG)2a, rat IgG2b, and hamster IgG1 isotype antibodies conjugated with the appropriate fluorochromes were used (BD Biosciences). The stained cell suspensions were analyzed using FACSCalibur or FACSCanto II for the percentage of positively stained cells, or for the mean fluorescence intensity.

### 2.7. Neutralization Assay

The neutralizing antibody titers from the immune sera were prepared using a series of two-fold dilutions in the cell culture medium—Dulbecco’s Modified Essential Medium (D-MEM), supplemented with 10% FBS, 100 U/mL penicillin, 100 µg/mL streptomycin, and 0.25 µg/mL amphotericin B. Approximately 20.5 PFU of HSV-2 strain 333 was added to each tube, containing the diluted serum, and to the control tubes, containing only the medium, in order to achieve a final volume of 600 μL. Following incubation at RT for 1 h, the HSV-2 PFU/mL in each tube was quantified by titration on Vero cell monolayers. The neutralizing titer was expressed as the percent of inhibition of the viral infection.

### 2.8. Quantitative Reverse Transcriptase-Polymerase Chain Reaction

The total RNA was isolated from the spinal cords and the vaginal tissues preserved in RNAlater (Sigma Aldrich) using a Universal RNA Purification Kit (Eurx). Transcripts of IFN-α, IFN-γ, CXCL9, CXCL10, and GAPDH were quantified using Taqman(R) Gene Expression Assays (Thermo Fisher Scientific). All of the PCR reactions were carried out with the QuantiFast Probe RT-PCR Kit (Qiagen, Hilden, Germany), using a real-time PCR instrument, Stratagene MX4000 Real-Time qPCR System (Agilent Technologies), according to the manufacturer’s protocol. The 2^−∆∆Ct^ method was used for calculating the relative ratio to the control uninfected tissue.

### 2.9. Statistical Methods

Data are presented as the mean ± standard error of the mean (SEM) from at least three independent experiments. The data were analyzed using Biostat 2009 software and the two-tailed paired Student’s *t*-test (normal distribution) or non-parametric Kruskal–Wallis and Wilcoxon tests. In every analysis, values of *p* ≤ 0.05 were considered significant.

## 3. Results

### 3.1. Characterization of AgNPs

**Figure 1:**
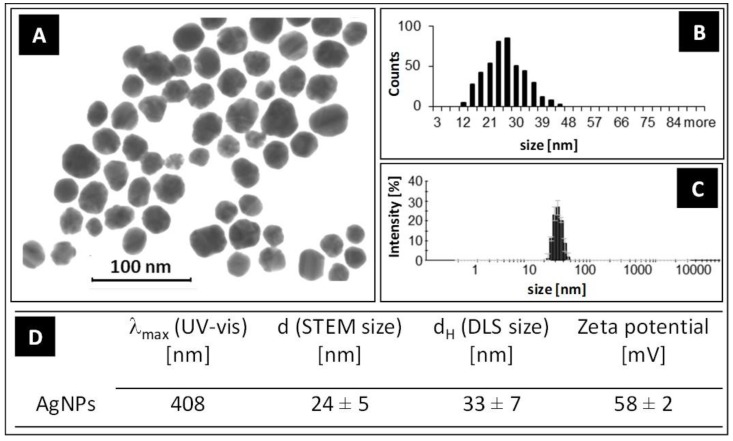
Scanning transmission electron microscopy (STEM) image of silver nanoparticles (AgNPs) (**A**), STEM size distribution histogram (**B**), dynamic light scattering (DLS) size distribution histogram (**C**), and the overall results of the AgNPs’ characterization (**D**).

The shape and size of nanoparticle metallic cores were determined with STEM technique (NovaNanoSEM 450 FEI equipped with STEM II detector for transmitted electron detection, acceleration voltage 30 kV, spot size 1.5). The hydrodynamic size and zeta potential of the nanoparticles (the size of a metallic core along with substances present on the nanoparticles surface) were measured using the DLS technique (Nano ZS Zetasizer system, Malvern Instruments, Worcestershire, U.K.), while the colloidal stability of the nanoparticles was determined using DLS, UV-VIS spectroscopy (Ocean Optics, HL-2000), and zeta potential measurements. The STEM image, DLS size distribution histogram, and the overall results of the TA-AgNPs characterization are presented in Figure 1. The size of a metallic core of silver nanoparticles was 24 ± 5 nm. The hydrodynamic size of the nanoparticles was 33 ± 7 nm. The shell of the stabilizers adsorbed on the NPs surface consisted of complexes of tannic acid and sodium citrate, which took part in the synthesis of NPs and its further stabilization in a colloidal solution [25]. The negative value of the zeta potential confirmed a high storage stability of the colloid (−58 ± 2 mV).

### 3.2. Virus-Nano Interaction

**Figure 2:**
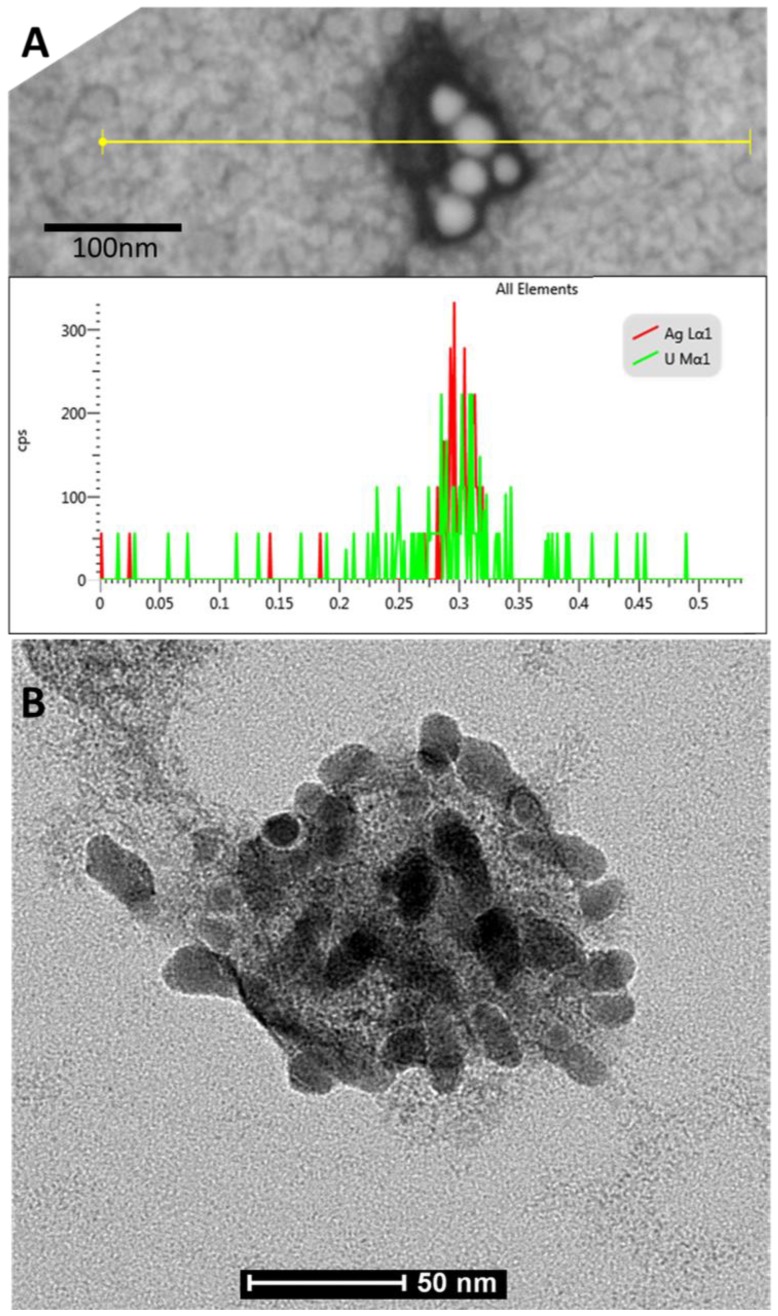
(**A**) The ultrastructure of Herpes simplex virus-2 (HSV-2) with attached several AgNP particles and EDX spectra of silver and uranium aligned with observed objects, acquired using the line-scan mode; scanning-transmission electron microscope, scale bar = 100 nm. (**B**) The ultrastructure of HSV-2 covered with silver nanoparticles; transmission electron microscope, scale bar = 50 nm.

The STEM analysis revealed the presence of virus-nanoparticles complexes, visible as round moderately contrasted objects of about 100 nm in diameter with highly contrasted oval objects of about 30 nm in diameter attached. The line-scan EDX analysis showed the prominent peak of silver (nanoparticles) and uranium (nucleic acid) aligned with observed objects (Figure 2A). The TEM observations revealed the presence of objects of about 100 nm in diameter, identified as HSV-2 viruses, covered with numerous nanoparticles of about 30 nm in diameter (Figure 2B).

### 3.3. TA-AgNPs-Treated Mice Show Better Clinical Scores

**Figure 3:**
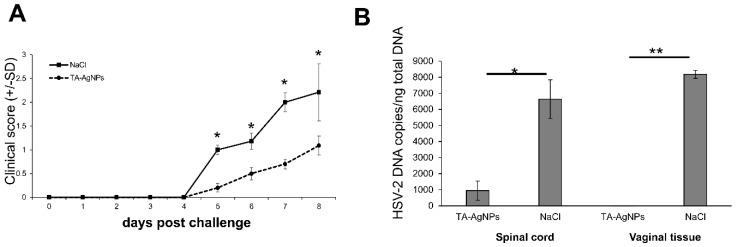
Tannic acid modified silver nanoparticles (TA-AgNPs) reduce genital HSV-2-infection in mice. C57BL6 mice were inoculated intravaginally with 10^3^ PFU of HSV-2, then treated with 5 µg/mouse of TA-AgNPs at 6, 24, and 48 h after infection. Disease development expressed as mean disease score (**A**); (n = 5–20). (**B**) The numbers of the HSV-2 DNA copies in the vaginal tissues obtained on day eight post-infection, and in the spinal cord obtained on day eight of infection were assessed by PCR. The means are expressed as mean ± standard error of the mean (SEM) for n = 20; * represents significant differences with *p* ≤ 0.05, while ** means *p* ≤ 0.01.

To study the ability of AgNPs to inhibit HSV-2 infection in vivo, we used a well-known mouse model of a genital HSV-2 infection. We have previously demonstrated that tannic acid modified silver nanoparticles (TA-AgNPs) block the infectivity of HSV-2, if inoculated intra-vaginally as a mixture of HSV-2 and TA-AgNPs [19]. Here, we investigated whether TA-AgNPs can help to inhibit the primary HSV-2 infection. To mimic the treatment of primary lesions, the female mice were first inoculated intra-vaginally with HSV-2, then treated by washing with 5 µg/mL TA-AgNPs/NaCl or NaCl at 6, 24, and 48 h after infection. The clinical scores showed significant differences in the TA-AgNPs-treated group, starting from day five post-infection (p.i.) in comparison witho the NaCl-treated group (Figure 3A). The TA-AgNPs-treated mice did not show neurological signs, and the vaginal inflammation was less pronounced (Figure 3A). This was reflected by significantly lower levels of viral DNA in the vaginal tissue at day eight p.i., observed in the TA-AgNPs-treated mice in comparison with the NaCl-treated mice (*p* ≤ 0.01) (Figure 3B), as well as in the spinal cords (*p* ≤ 0.05) (Figure 3B).

### 3.4. TA-AgNPs Help to Mount Early Immune Response

In our previous work, we demonstrated that TA-AgNPs activate dendritic cells in vitro [21], but also modulate the local inflammatory response when applied together with HSV-2 [19]. Here, we investigated whether TA-AgNPs can contribute to the activation of early immune response. The cell suspensions prepared from the vaginal tissues were subjected to immunophenotyping for CD4+ T cells, CD8+ T cells, NK cells, CD4+ CD69+ T cells, CD8+ CD69+ T cells, dendritic cells, and monocytes (Table 1). Treatment with TA-AgNPs early after infection led to a significant increase in the percentages of dendritic cells and monocytes at day eight p.i. (*p* ≤ 0.05) (Table 1). The vaginal tissues treated with TA-AgNPs also showed an increase in the percentages of all of the types of T cells and NK cells, albeit the differences were insignificant (*p* ≤ 0.05) (Table 1).

### 3.5. Treatment with TA-AgNPs Helps to Boost Anti-Viral Immunity

**Figure 4:**
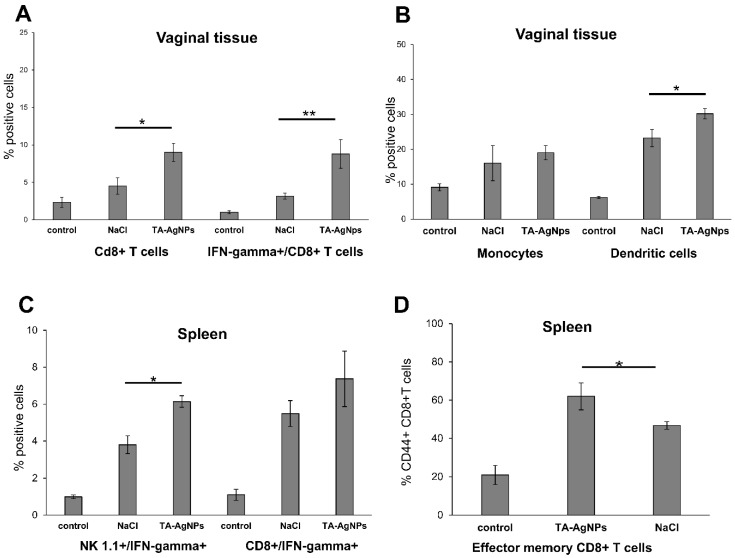
Treatment with TA-AgNPs helps to boost T and NK cells’ response after re-challenge. C57BL6 mice were infected intravaginally with HSV-2, then treated with 5 µg/mouse of TA-AgNPs 6, 24, and 48 h after infection. Thirty days later, the mice were re-challenged with the same virus dose. The cell suspensions from the vaginal tissues and spleens were prepared 10 days after re-challenge. The percentage of CD8+ T cells and CD8+/IFN-gamma+ T cells (**A**), and monocytes and dendritic cells (**B**) in the vaginal tissue, as well as the percentage of NK1.1/IFN-gamma+ cells and CD8+/IFN-gamma+ T cells (**C**), and the effector memory CD8+ T cells (**D**) were accessed by flow cytometry. The means are expressed as mean ± SEM for n = 20; * represents significant differences with *p* ≤ 0.05, while ** means *p* ≤ 0.01.

We previously observed that HSV-2 treated with TA-AgNPs was a good stimulator of memory T cells in co-cultures of antigen-specific T cells and dendritic cells [21]. Here, we investigated whether a post-infection treatment with TA-AgNPs can help to activate an anti-HSV-2 response upon being re-challenged with the virus. The mice treated with NaCl or TA-AgNPs early after infection were re-challenged with the same virus dose 30 days after the primary infection, and were sacrificed at day 10 after re-challenge, in order to obtain the serum and the vaginal tissue. We found that treatment with TA-AgNPs sonon after infection led to a significant increase in the percentages of CD8+ T cells and IFN-gamma+ CD8+ T cells in the cell suspensions prepared from the vaginal tissues 10 days after the re-challenge (*p* ≤ 0.05) (Figure 4A). Furthermore, the percentage of dendritic cells was significantly up-regulated after re-challenge in the TA-AgNP treated vaginal tissues (*p* ≤ 0.05) (Figure 4B). An assessment of the IFN-gamma producing cells in the spleens isolated 10 days after re-challenge with HSV-2, showed that the TA-AgNPs treatment led to a significantly higher percentage of IFN-gamma producing NK cells and effector-memory CD8+ T cells (*p* ≤ 0.05) (Figure 4C,D).

Furthermore, we measured the levels of cytokines and chemokines important in raising the anti-HSV-2 antiviral response, namely IFN-alpha, IFN-gamma, CXCL9, CXCL10, and CXCL17 (Table 2), in the vaginal tissues 10 days after re-challenge. In general, we have not found significant differences in the mRNA expressions of the selected cytokines and chemokines, except for CXCL17 (Table 2). The expression of this cytokine was very strongly up-regulated in the vaginal tissues treated with TA-AgNPs during primary infection (*p* ≤ 0.05) (Table 2).

**Figure 5:**
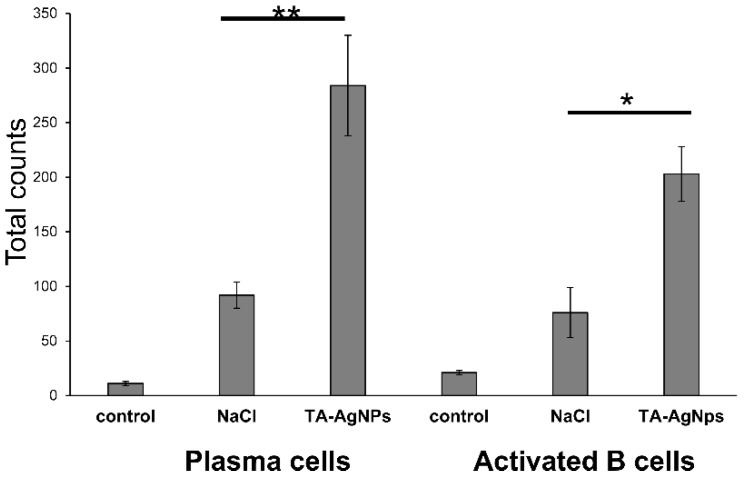
Treatment with TA-AgNPs helps to boost B cells’ response after re-challenge in the vaginal tissue. C57BL6 mice were infected intravaginally with HSV-2, and treated with 5 µg/mouse of TA-AgNPs 6, 24, and 48 h after infection. Thirty days later, the mice were re-challenged with the same virus dose. The cell suspensions from the vaginal tissues prepared 10 days after re-challenge with HSV-2 were stained for plasma B cells (CD138+/B220low+/IgD−) and activated B cells (CD27+/B220+/IgD+). The means are expressed as mean ± SEM for n = 20; * represents significant differences with *p* ≤ 0.05, while ** means *p* ≤ 0.01.

**Figure 6:**
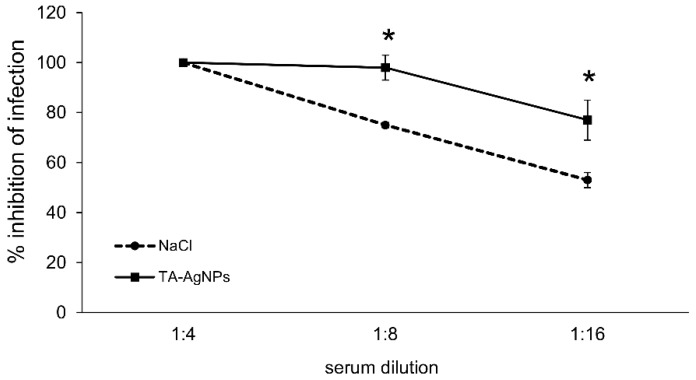
Intravaginal treatment with TA-AgNPs of HSV-2 infection induces better seroneutralization titers. The C57BL6 mice were infected intravaginally with HSV-2, and treated with 5 µg/mouse of TA-AgNPs, then re-challenged thirty days later with the same virus dose. Sera were taken at 10 days after re-challenge with HSV-2, and were subjected to seroneutralization tests. The means are expressed as mean ± SEM for n = 20; * represents significant differences with *p* ≤ 0.05.

We next assessed whether the TA-AgNP treatment after infection could confer an anti-viral protective antibody response. The animals from the TA-AgNP treated group showed significantly higher counts of plasma cells and activated B cells in the vaginal tissue in comparison with the NaCl-treated group (*p* ≤ 0.05) (Figure 5), while no significant differences were shown for the memory B cells (data not shown). Functional HSV-2-neutralizing antibodies were produced by both the NaCl and TA-AgNP treated mice, although the TA-AgNP treated animals showed significantly better titers of anti-HSV-2 neutralization antibodies in sera (*p* ≤ 0.01) (Figure 6).

## 4. Discussion

Hydrolysable tannins (HTs) are the secondary metabolites from plants, with molecular weights ranging from 500 to 30,000 Da [26]. They are classified into gallotannins and ellagitannins, with gallic acid and ellagic acid units, respectively, attached to the hydroxyl group of glucose by an ester linkage [25]. Tannic acid (penta-m-digalloyl glucose) is a hydrolysable tannin, well-known for its anti-oxidative, anti-inflammatory, and antiviral properties [11,12,26,27,28]. It has been demonstrated that the polyphenolic nature of tannic acid (hydrophobic core and hydrophilic shell) allows for its interaction with cellular surface proteins [29]. Tannic acid has been shown to inhibit the attachment of viruses to host cells, including norovirus binding to HBGA receptors and the attachment of the influenza A virus to Vero and Madin-Darby canine kidney cells, as well as blocking the hepatitis C virus entry into Huh7.5 cells [11,12,30]. Other tannins, such as epigallocatechin-3-gallate (EGCG), chebulagic acid, and punicalagin have been suggested to possess a broad-spectrum of antiviral activities, including the inactivation of sexually transmitted viruses such as HIV and HSV-2 [31,32,33]. Furthermore, they were also effective against other viruses using glycosaminoglycans for entry—human cytomegalovirus (HCMV), hepatitis C virus (HCV), dengue virus (DENV), measles virus (MV), and respiratory syncytial virus (RSV) [32,33].

Although the mechanisms of the polyphenols’ antiviral actions have been primarily linked with their antioxidant activity, the ability of polyphenols to prevent the entry of viruses into cells is actually due to their ability to form macromolecular complexes on the cell surface [29,33,34]. The superior efficiency of TA-AgNPs at viral inactivation might also depend on the proline content in HSV glycoproteins. Luck et al. (1994) demonstrated that tannic acid moieties easily interact with proline-rich proteins [35]. The pro-fusion domain of the HSV glycoprotein D, which was demonstrated to be crucial for triggering the signaling pathways and membrane fusion at viral entry, is particularly rich in proline residues [36]. We have previously shown that, although tannic acid is able to block the entry and penetration of HSV-2 into keratinocytes, in combination with silver nanoparticles, it boosts its anti-viral properties several times [19]. The interaction between tannic-acid modified silver nanoparticles and the virus surface in vitro is much faster than with tannic acid alone [19]. Furthermore, TA-AgNPs applied both as a colloid or in an appropriate mucoadhesive gel efficiently inhibit HSV-2 infection in the mouse model [19,20]. However, the antiviral properties of TA-AgNPs depend strongly on the size of the nanoparticles, with 20–40 nm silver nanoparticles being the most effective, without undesired toxic and pro-inflammatory properties when applied in vivo [19].

As we discussed previously, the toxicity of NPs modified with tannic acid can vary with the cell type, cellular uptake, and size [19,22]. Here, we used TA-AgNPs of 33 nm, which show moderate toxicity in vitro, do not induce inflammatory responses [19,22], and are still good stimulators of dendritic cells [21]. Most NPs tend to aggregate in biological solutions, increasing their overall size. Thus, we cannot exclude the possibility that tannic acid-modified NPs used as a microbicide may form aggregates in biological fluids, such as vaginal fluid. Furthermore, the aggregates of tannic acid-modified NPs with viral antigens may be better internalized and processed by antigen presenting cells (APCs), thus leading to an improved activation of immune competent cells downstream. We hypothesize that the combination of tannic acid and silver nanoparticles constitutes an effective anti-viral microbicide to be applied on mucosal tissues, with additional adjuvant properties boosting its anti-viral response. For HSV-2, the pathway to antigen presentation is complex, involving multiple types of DCs. The virus first infects LCs, which undergo apoptosis and the apoptotic bodies are taken up by bystander DCs. The activated migratory DCs carry HSV antigens out of the infected tissue and are essential for T-cell priming in draining lymph nodes [37]. However, we have noticed that the HSV-2 infection of DCs inhibits their maturation and migration, and thus results in a poor development of the specific immune response [38]. Also, inactivated HSV-2 has been shown to be a poor antigen for immature DCs, irrespective of the inactivation mode [38]. Here, we found that TA-AgNPs stimulate the migration of DCs into the vaginal tissue, not only early during primary infection, but also later, upon re-challenge with the virus. This may help to present the virus antigens both to naïve cytotoxic T cells in the lymph nodes and to memory T cells, resulting in the activation of the specific anti-viral response. We previously showed that co-cultures of antigen-specific T cells with dendritic cells stimulated with NP-treated HSV-2 led to a significant increase in the proliferation of CD8+ IFN-γ+ T cells, in comparison with live or inactivated HSV-2 [21]. In this study, we also observed a significantly higher infiltration of CD8+ T cells and IFN-gamma+ CD8+ T cells into the vaginal tissue after treatment with TA-AgNPs, in comparison with NaCl treatment. Furthermore, we also detected a significantly higher percentage of effector memory CD8+ T cells in the spleens of TA-AgNP treated animals. Previously, we showed that HSV-2 treated with TA-AgNPs was a better stimulant of the effector memory CD8+ T cells in comparison with inactivated HSV-2 and live HSV-2 [21]. Interestingly, treatment with TA-AgNPs early during the HSV-2 infection induced a strong up-regulation of CXCL17 chemokine in the vaginal tissue later, after re-challenge. Srivastava et al. (2018) have recently demonstrated that the CXCL17/CXCR8 chemokine pathway plays a crucial role in mucosal vaginal immunity by the mobilization of the functional protective CD8+ effector and resident memory T cells, within this site of acute and recurrent HSV-1 infection [39]. Our findings correlate with this study and may support the role of this chemokine in mucosal immunity.

Many studies have been focused on the T cell-mediated control of HSV infections. For example, several studies using experimental models showed that immunity to HSV was conferred upon naïve mice by the adoptive transfer of CD4+ and/or CD8+ T-cells specificly for particular HSV antigens [40,41]. However, vaccine-induced memory T-cell responses require time for activation and for delivery to the HSV-2 infected sites. In contrast, antibodies are pre-formed and present in mucosal secretions, and are ready to inhibit the viral spread immediately upon exposure. The results of the study by Halford et al. (2015) provide clear proof that HSV-specific antibodies also have a critical role in protective immunity to HSV-2 [42]. Vaccination with a live HSV-2 ICP0-mutant vaccine induced equivalent CD8+ T cell responses in wild-type and B-cell deficient mice [42]. However, the vaccinated B-cell deficient mice shed ~40-fold more HSV-2 virions 24 h post-challenge in comparison with the vaccinated wild-type (B-cell+) mice, and eventually died [41].

McDermott et al. [43] and Milligan and Bernstein [44] first demonstrated immunoglobulin G (IgG) antibodies specific for HSV-2 in vaginal secretions, while neutralizing IgA antibodies were not detected or were detected at very low titers. The migration and homing of the plasma cell precursors from the blood is important for the anti-HSV-2 response [45]. Intravaginal immunization against HSV-2 is much more effective than intranasal immunization in terms of higher IgG titres, higher plasma cell numbers, and higher memory T-cell responses to the challenge, as well as better protection against the challenge infection [46].

Here, we showed that treatment with anti-viral microbicide in the form of tannic acid-modified silver nanoparticles of the HSV-2 infected vaginal tissue elicited B cells’ activation and plasma cells’ homing. Furthermore, treatment with TA-AgNPs also provided a systemic protection, suggesting that TA-AgNPs help to carry HSV antigens into the draining lymph nodes, where they can be recognized by naïve B cells, and can activate them to develop long-term anti-HSV-2 memory.

Taking into account the use of tannic acid modified AgNPs as microbicides, we can therefore hypothesize that upon treatment of the primary infection, the HSV-2 virions treated with tannic acid modified silver nanoparticles may be more effectively internalized by migratory DCs, activate DCs to present and prime CD4+ and CD8+ T cells, and also to effectively activate B cells. During secondary, recurrent infections, NP treated HSV-2 antigens can effectively activate antigen specific memory T cells, but also antigen specific memory B cells, which are present in the mucosal tissue. To our knowledge, our results provide the first evidence that an anti-HSV microbicide constructed from tannic acid modified silver nanoparticles may provide an improvement in the specific immune response to recurrent infections. We showed here that treatment with TA-AgNPs helps to develop a virus-specific cellular and humoral response, which is effective upon re-challenge. This is of great importance, as patients infected latently with HSV-1 or -2 usually experience two to three recurrent infections during the year. Therefore, the treatment of both a primary and recurrent infection with TA-AgNPs could boost a virus-specific cellular and humoral response, helping the immune system to limit the virus spread and reappearance from the sites of latent infection.

As there are many tannins with defined anti-viral properties, in particular against herpesviruses, we can speculate that our results may provide data for preliminary studies on tannin-modified, nano-metal-based classes of microbicides with adjuvant properties.

## Figures and Tables

**Table 1:** Percentage of CD4+ T cells, CD8+ T cells, NK cells, CD4+ CD69+ T cells, CD8+ CD69+ T cells, dendritic cells, and monocytes in the vaginal tissue treated with NaCl or silver nanoparticles with tannic acid (TA-AgNPs) at 6, 24, and 48 h post-infection (p.i.) and isolated for further assays after eight days p.i. The results are expressed as mean ± standard error of the mean (SEM) for n = 10. and * = *p* < 0.05. HSV-2—herpes simplex virus 2

Control (Uninfected)	HSV-2 + NaCl Treatment	HSV-2 + TA-AgNP Treatment
CD4+ T cells		
2.5 ± 0.5	14.89 ± 3.52	19.2 ± 4.7
% of CD69+ CD4+ T cells		
8.8 ± 1.1	40.2 ± 4.24	47.6 ± 2.4
CD8+ T cells		
2.12 ± 0.45	11.15 ± 2.25	13.8 ± 4.48
% of CD69+ CD8+ T cells		
9.2 ± 2.1	52.2 ± 6.12	49.4 ± 4.05
NK cells		
2.54 ± 0.67	9.82 ± 2.62	9.4 ± 2.69
Monocytes		
8.34 ± 0.9	18.8 ± 1.1	25.4 ± 2.8 *
Dendritic cells		
5.41 ± 1.2	16.4 ± 0.67	26.8 ± 2.7 *

**Table 2:** Cytokine and chemokine expression changes in the vaginal tissues at 10 days after re-challenge with HSV-2. The vaginal tissues were earlier subjected to treatment with TA-AgNPs during primary HSV-2 infection. The mRNA levels of the IFN-alpha, IFN-gamma, CXCL9, CXCL10, and CXCL17 are shown as an expression relative to the control, on the basis of the 2^−∆∆Ct^ method. The mRNA levels were calculated from three PCR reactions for each sample. n = 10. ** *p* ≤ 0.001 versus NaCl-treatment.

HSV-2 + NaCl Treatment	HSV-2 + TA-AgNPs Treatment
*IFN-alpha*	
8192 ± 54	8902 ± 67
*IFN-gamma*	
25 ± 4.3	19.42 ± 5.1
*CXCL9*	
430 ± 51	317 ± 56
*CXCL10*	
1243 ± 121	1024 ± 99
*CXCL17*	
2048 ± 128	33,454,432 ± 53 **
